# Medicaid Analysis of Substances of Abuse for Patients With Crohn’s Disease

**DOI:** 10.1016/j.gastha.2023.07.015

**Published:** 2023-07-25

**Authors:** Sara E. Yacyshyn, Bruce R. Yacyshyn

**Affiliations:** Wright State University, Boonshoft School of Medicine, Dayton, Ohio; Department of Pharmacology and Systems Physiology, University of Cincinnati School of Medicine, Cincinnati, Ohio

Crohn’s disease (CD) is a chronic inflammatory bowel disease (IBD) of the gastrointestinal tract with heterogenous symptoms. The symptom of pain is often one of the most debilitating yet variably addressed.^1^ Recent advances in the understanding of the mechanisms of inflammation of IBD have led to progress in the drug-targeting capabilities of treatments. However, like many chronic diseases, the subjective nature of pain and the confounding overshadowing influence of psychological stress make it one of the most challenging symptoms.^1^ Substance abuse is an unfortunate, yet common, path chosen by some symptomatic patients. Partially accounting for this reaction, is the median young age of afflicted patients (29.5 years).^2^^,^^3^ This is an age-based demographic group particularly vulnerable to recent societal events and thus, has experienced increased rates of related medical complications.^4^ From a pharmaco-economic perspective, the impact of abdominal pain in CD as a marker for substance abuse is also associated as a cost-driver.^5^

Abdominal pain is commonly reported in patients with CD and is closely associated with reduced quality of life.^6^ Even with apparent remission of inflammation, approximately 41% of patients with CD still experience irritable bowel syndrome-like symptoms, including pain, bloating, or erratic bowel habits.^7^ In many cases, pain is severe enough to warrant pain-specific treatment, but current treatment options are often insufficient. Often, this is a result of the absence of availability of gut-specific analgesics. The treatment of abdominal pain in many chronic conditions includes over the counter analgesics and nonsteroidal antiinflammatory drugs, antispasmodics, antidepressants, and opioids.^8^^,^^9^ Non-steroidal anti inflammatory drugs are associated with the increased risk of gut mucosal damage and ulceration, and induction of irritable bowel syndrome flares.^10^ Similarly, alcohol use has been associated with flares of IBD symptoms due to its prooxidant characteristics and impact on gut barrier function.^11^ Cannabis use has been reported in patients with IBD, however, as a pharmacologic agent has not proven to be a particularly effective analgesic, reflecting the equivocal clinical data in IBD.^12^, ^13^, ^14^

In this current issue of Gastro Hep Advances, Chen et al^15^ used Medicaid data from 2010 to 2019 in their well-designed observational study to evaluate substance abuse amongst patients with CD to help answer questions relating to ascertaining the “size and circumstance of the problem [of substance use in a CD patient population].” They used national Medicaid databases from 2010 to 2019 to identify participants with newly diagnosed CD and defined substance use (which included alcohol, opioids, cocaine, amphetamines, and cannabis). Multivariable logistic regression models were performed to determine the associations between CD-related interventions and substance use before/after CD diagnosis and hospitalizations. Of their 37,323 Medicaid recipients, the authors noted 16.3% (6091) of patients with ever-use of the specific substances listed. Of note, 54.1% of users were women, and 64.3% were non-Hispanic White.

Consideration of the observed association of exposure (hospitalization) and outcome (alcohol use after diagnosis) was analyzed. Interestingly, the any-substance use cohort decreased after diagnosis. The authors did note that substance-specific consumption was largely stable, with opiates increasing and alcohol decreasing after diagnosis of CD. Subgroup analysis did show CD-related hospitalization (not surgery) and steroid use subsequently caused increased use of alcohol. CD specific surgery was noted to have lower opioid misuse after, unlike steroid use which associated with increased opiate use. Surprisingly, cannabis and amphetamines were least commonly used among the 5 substances surveyed. Although a higher rate of substance use was found in patients with CD then the general US population, the rate of one component of this analysis showed that cannabis use was lower than the general population. The etiology of this discrepancy is speculated as economic, with alcohol and cannabis use reported as higher for higher income groups than for Medicaid recipients. Perhaps this discrepancy may arise from the lack of analgesic efficacy of cannabis, with patients seeking more effective methods of pain management.

The findings in this study are complex and need to be viewed as a slice of a larger pie in which resources to purchase substances of self-medication are available. However, this paper does seem to support the premise that improvement in health status results in lower use of illegal substances in CD for symptom management. Albeit the etiology of those drivers is not readily apparent in the analysis. Further research is needed for nonaddictive, noncognitively impairing strategies for pain and mental health management, such as potentially offered by CB2 selective agonists such as olorinab.^16^

In depth analysis is also important in planning for resource allocation for chronic illnesses in North America given the persistence of IBD. Moreover, individual IBD patient care should include mental health and substance abuse screening.

## References

[1] Singh S., Blanchard A., Walter J.R. (2011). Common symptoms and stressors among individuals with inflammatory bowel diseases. Clin Gastroenterol Hepatol.

[2] Loftus E.V., Shivashankar R., Tremaine W.J. (2014). ACG 2014 annual scientific meeting.

[3] Bogale K., Vrana K., Raup-Konsavage W. (2021). Polysubstance abuse in inflammatory bowel disease. Dig Dis.

[4] Alkouri N., Almomani A., Le P. (2022). The prevalence of alcoholic and nonalcoholic fatty liver disease in adolescents and young adults in the United States: analysis of the NHANES database. BMC Gastroenterol.

[5] Fatakhova K., Patel P., Inayat F. (2023). Trends in hospital admissions and mortality among inflammatory bowel disease patients with substance use disorder: a 10-year United States nationwide analysis. Proc (Bayl Univ Med Cent).

[6] Zeitz J., Ak M., Muller-Mottet S. (2016). Pain in IBD patients: very frequent and frequently insufficiently taken into account. PLoS One.

[7] Halpin S.J., Ford A.C. (2012). Prevalence of symptoms meeting criteria for irritable bowel syndrome in inflammatory bowel disease: systematic review and meta-analysis. Am J Gastroenterol.

[8] Srinath A.I., Walter C., Newara M.C. (2012). Pain management in patients with inflammatory bowel disease: insights for the clinician. Therap Adv Gastroenterol.

[9] Benyamin R., Trescot A.M., Datta S. (2008). Opioid complications and side effects. Pain Physician.

[10] Klein A., Eliakim R. (2010). Non steroidal anti-inflammatory drugs and inflammatory bowel disease. Pharmaceuticals.

[11] Swanson G.R., Sedghi S., Farhadi A. (2010). Pattern of alcohol consumption and its effect on gastrointestinal symptoms in inflammatory bowel disease. Alcohol.

[12] Mantzouratis G., Fafliora E., Saridi M. (2018). Alcohol and narcutics use in inflammatory bowel disease. Ann Gastroenterol.

[13] Naftali T., Bar-Lev Schleider L., Dotan I. (2013). Cannabis induces a clinical response in patients with Crohn's disease: a prospective placebo-controlled study. Clin Gastroenterol Hepatol.

[14] Bicket M.C., Stone E.M., McGinty E.E. (2023). Use of cannabis and other pain treatments among adults with. chronic pain in theUS states withMedical cannabis programs. JAMA Netw Open.

[15] Chen P.-H., Patel R., Miller S.D. (2023). Substance use among patients with incident Crohn’s disease in the United States, 2010 to 2019: a medicaid observational study. Gastro Hep Advances.

[16] Yacyshyn B.R., Hanauer S., Klassen P. (2020). Safety, pharmacokinetics, and efficacy of Olorinab, a peripherally acting, highly selective, full agonist of the cannabinoid receptor 2, in a phase 2a study of patients with chronic abdominal pain associated with Crohn's disease. Crohns Colitis 360.

